# Exogenous Melatonin Application Enhances Pepper (*Capsicum annuum* L.) Fruit Quality via Activation of the Phenylpropanoid Metabolism

**DOI:** 10.3390/foods14071247

**Published:** 2025-04-03

**Authors:** Feibiao Gao, Kangning Han, Weilan Ma, Jing Zhang, Jianming Xie

**Affiliations:** College of Horticulture, Gansu Agricultural University, Lanzhou 730070, China; gaofb1001@163.com (F.G.); hankn0204@126.com (K.H.); 15593551366@163.com (W.M.)

**Keywords:** *capsicum annum* L., melatonin, phenolic compounds, enzymatic activity, gene expression

## Abstract

Melatonin (MT) is an elicitor that stimulates phenolic compounds biosynthesis and accumulation in fruits and vegetables. However, its role in regulating phenolic compounds and the phenylpropane metabolism during pepper ripening is unclear. To investigate how exogenous MT regulates phenolic compounds biosynthesis during pepper ripening, pepper plant surfaces were sprayed with different MT concentrations (0 and 100 µmol·L^−1^) 10 days after anthesis. MT treatment improved pepper fruits quality. In particular, total phenolics and flavonoids compounds levels were elevated, indicating that MT affected phenolic compounds metabolism. Furthermore, metabolomics identified 15 substances exhibiting high fold-change values after MT treatment, including chlorogenic acid, gallic acid, ferulic acid, caffeic acid, cynarin, *p*-coumaric acid, cinnamic acid, gentianic acid, benzoic acid, sinapic acid, *p*-hydroxybenzoic acid, protocatechuic acid, rutin, quercetin, and kaempferol. Shikimate dehydrogenase, phenylalanine ammonia-lyase, cinnamate-4-hydroxylase, 4-coumarate-Coa ligase, chalcone synthase, and chalcone isomerase activities were also evaluated. MT upregulated the expression of genes involved in phenolic compounds synthesis during pepper ripening and that of corresponding genes involved in the endogenous MT anabolic pathway, promoting endogenous. The polyphenolics and carbohydrates are indicators of the botanical and geographical origin of Serbian autochthonous clones of red spice MT synthesis throughout pepper ripening. In summary, exogenous MT accelerates phenolic compounds synthesis in pepper fruits by activating phenylpropane metabolism and modulating endogenous hormone signaling networks. This is expected to offer a revolutionary strategy to reinforce pepper plants resistance and quality.

## 1. Introduction

Peppers (*Capsicum annuum* L.), members of the nightshade family (Solanaceae), are globally cultivated as a major vegetable and spice crop, renowned for their pungent fruits [1]. This species exhibits significant genetic diversity and is rich in health-promoting chemical compounds, such as capsaicinoids, carotenoids, phenols (flavonoids and phenolic acids), vitamins (C and E), and minerals, with anticancer, anti-inflammatory, antimicrobial, and antioxidant properties [2]. With improvements in living standards and an emphasis on health, the demand for quality fruits and vegetables is rapidly increasing [3]. Facility-grown pepper, comprising one of the main vegetables cultivated during the winter and spring seasons, is favored by growers and consumers owing to its advantages, such as its off-season production, short growth cycles, and high efficiency. Temperature and light are important ecological factors that affect the yield and quality of pepper [4]. However, in northern China, where pepper is cultivated in overwintering facilities, extreme weather conditions, such as low temperature, low light, and high humidity, frequently occur. These environmental stresses negatively affect pepper production, disrupt the synthesis of phenolic compounds, and ultimately affect their quality and marketability, thereby hindering the efficient cultivation and off-season production of pepper. Therefore, improving cultivation technology to enhance the quality of pepper is of great significance in guaranteeing the sustainable development of the facility-cultivated pepper industry.

Melatonin (MT), an indole molecule widely found in animals and plants, is an endogenous metabolite that can be degraded by organisms themselves [5]. It is non-toxic, safe, and non-residual in the human body [6]. MT is well known for enhancing plant resistance to various biotic and abiotic stresses, owing to its potent free radical-scavenging ability, while also modulating plant signaling and response pathways through mechanisms that are largely unknown [7,8]. Recently, increasing studies have shown that MT can play a crucial role in improving crop quality and yields by regulating various aspects of plant growth and development [9,10,11]. Furthermore, recent studies have shown that it is an important technological tool for increasing the accumulation of phenolic compounds in fruits and vegetables, such as cherries [12], litchis [13], raspberries [14], and cabbages [15]. Although numerous studies have explored the effects of MT on plant growth, development, and adaptation, information on the effect of exogenous MT on the regulation of phenolic compounds and the phenylpropane metabolism during pepper ripening is limited. In the present study, changes in phenolic compounds, related enzymatic activities, and genes expression during the ripening of pepper treated with exogenous MT were analyzed to provide valuable insights into the use of MT as a tool to regulate the phenylpropanoid pathway during pepper ripening.

## 2. Materials and Methods

### 2.1. Materials and Experimental Design

**Figure 1:**
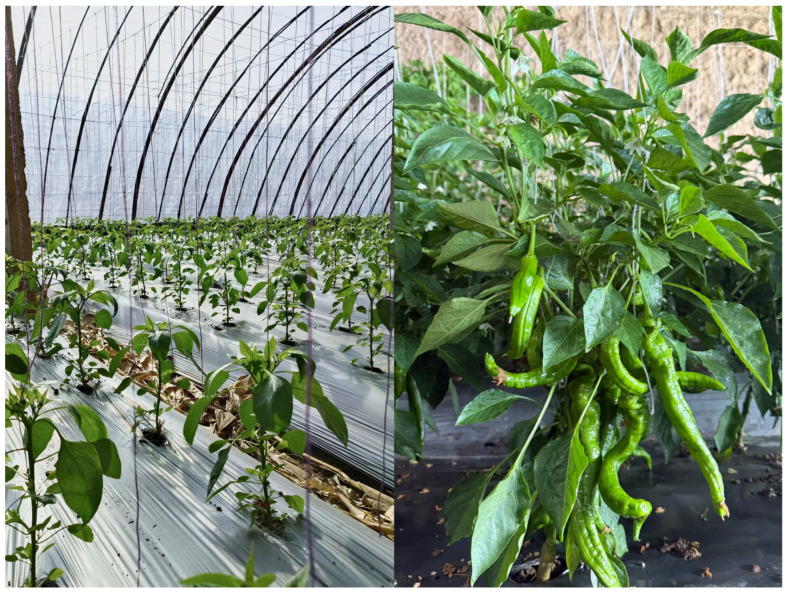
The growth and development of pepper. Seedling stage (**left**) and maturity (**right**).

The experimental pepper plants (Huamei 105) were cultivated and harvested in a solar greenhouse located in Jingyuan County, Baiyin City, China (36.57 N, 104.68 E). The growth and development of pepper are illustrated in Figure 1. Two treatments were set up for this experiment, as follows: control (deionized water, containing 0.1% Tween-20 and 0.1% ethanol) and MT (100 μmol·L^−1^ MT aqueous solution, containing 0.1% Tween-20 and 0.1% ethanol). The optimal MT concentration was obtained from the results of previous experiments. During the experimental treatments, we labeled 40 healthy plants per treatment and used three replicates for a total of 120 plants. The third cluster fruit of plants with the same flower-opening date was selected, and MT was administered in the evening on days 10, 16, 22, 28, and 34 after anthesis. During this period, exogenous substances were sprayed onto the entire plant until the surface of the leaves were covered with water droplets. For sampling, 30 fruits from each treatment were randomly selected at 9:00 a.m. on days 16, 22, 28, 34, and 40 (green ripe stage) after anthesis, with three replicates per treatment. The collected samples were immediately fully frozen in liquid nitrogen and then stored at −80 °C for the measurement of relevant indicators.

### 2.2. Determination of Total Phenolics and Total Flavonoids Contents

Frozen fresh tissue (1 g) mixed with 10 mL of 1% HCl-methanol solution was maintained in the dark at 4 °C for 24 h to ensure thorough extraction. Subsequently, the resulting mixture was centrifuged at 12,000× *g* for 20 min at 4 °C. The supernatant was collected to determine the total phenolics and flavonoids contents. Total phenolics content was determined using a slightly modified version of the method described by Bai et al. [16]. Specifically, 0.5 mL of the extract was combined with 2 mL of Folin-Ciocalteu reagent and 2 mL of 7.5% (*w*/*v*) sodium carbonate (Na_2_CO_3_) solution. The mixture was then incubated at 50 °C for 5 min, and absorbance was measured at 760 nm. Total flavonoids content was analyzed following the method reported by Zhou et al. [17]. with little modifications. A reaction mixture containing 3.5 mL of the supernatant, 0.25 mL of 5% (*w*/*v*) sodium nitrite (NaNO_2_), and 0.25 mL of 10% (*w*/*v*) aluminum chloride (AlCl_3_) was prepared. After a 5 min incubation period, 1 mL of 1 M sodium hydroxide (NaOH) was introduced, and absorbance was then measured at 510 nm. The total phenolics and total flavonoids contents were quantified utilizing standard calibration curves prepared with gallic acid and rutin, respectively.

### 2.3. Determination of Phenolic Compounds Contents

Initially, 0.1 g of lyophilized fruit powder was accurately weighed into a centrifuge tube, mixed with 2 mL of 80% (*v*/*v*) methanol, and incubated at 4 °C for 1 h. During extraction, the mixture was continuously agitated to ensure thorough compounds dissolution. Subsequently, the samples underwent centrifugation at 12,000× *g* for 20 min at 4 °C. The supernatant was then filtered through a 0.22 µm organic-phase membrane into a brown bottle pending further determination. Referring to the research methodology proposed by Jin et al. [18], the conditions for HPLC were identified, and a symmetry C18 column (250 mm × 4.6 mm, 5 µm; Waters Corp, Milford, MA, USA) was utilized for HPLC separation, maintained at 30 °C, with an injection volume of 10 µL. The mobile phase consisted of methanol (A) and 1% (*v*/*v*) acetic acid (B), delivered at a flow rate of 1.1 mL min^−1^. Fifteen different compounds were detected at different wavelengths (including 322, 280, and 240 nm), as presented in (Table 1). The compounds were identified according to the retention time of the standard and quantified according to the standard curve.

### 2.4. Determination of Endogenous MT Content

Endogenous melatonin content was analyzed through a methodology slightly adapted from the research of Wu et al. [19]. Briefly, frozen tissue samples (0.5 g) were homogenized to a fine powder in liquid nitrogen and suspended in 5 mL of methanol. The suspension was sonicated at 80 Hz for 3 min at 4 °C, followed by centrifugation at 10,000× *g* for 15 min at 4 °C. The collected supernatant was subsequently dried under a gentle nitrogen stream. The residue was reconstituted in 2 mL of 50% (*v*/*v*) methanol and purified using a C18 solid-phase extraction (SPE) column (Waters, Milford, MA, USA). Prior to sample loading, the SPE column was activated sequentially with 5 mL methanol and 5 mL ultrapure water. The MT extract (2 mL) was loaded onto the column, washed with 10 mL of 5% (*v*/*v*) methanol, and eluted with 2 mL of 80% (*v*/*v*) methanol at a flow rate of 1 mL min^−1^. The eluate was filtered through a 0.22 μm nylon membrane and analyzed utilizing an Agilent 1290 Infinity liquid chromatography system coupled to a 6460 triple quadrupole mass spectrometer (LC-MS/MS; Agilent Technologies, Santa Clara, CA, USA). Chromatographic separation was accomplished using a mobile phase of acetonitrile (chromatographic grade) and 5 mM ammonium formate, delivered at a flow rate of 0.3 mL min^−1^. The injection volume was set at 5 μL were utilized. Detection of MT was facilitated at excitation/emission wavelengths of 280/348 nm, respectively. The melatonin content in the sample was subsequently calculated by comparing the peak area with that of the MT standard curve.

### 2.5. Determination of Enzyme Activities Involved in the Phenylpropanoid Metabolism Pathway

The shikimate dehydrogenase (SKDH) was assayed following the methodology detailed in Gao et al. [20]. Flesh tissue (1.0 g) was homogenized in 10 mL of 50 mM potassium-phosphate buffer (pH 6.8) and centrifuged at 12,000× *g* for 15 min at 4 °C, with the supernatant obtained for enzyme activity assays. The reaction mixture contained 1.9 mL of 100 mM Tris–HCl (pH 9.0), 1.45 mL of 2 mM shikimic acid, 1.45 mL of 0.5 mM NADP, and 0.2 mL of supernatant. The absorbance was monitored over 3 min by tracking the reduction of NADP at 340 nm. The activity of SKDH was expressed on a fresh weight basis as U·g^−1^, where U = 0.01 ΔA_340_ nm per minute.

Phenylalanine ammonia-lyase (PAL) activity was assayed by the protocol reported by Zhou et al. [21]. with minor modifications. Fresh tissue samples (1.0 g) were homogenized in 10 mL of 100 mM borate buffer (pH 8.8), which contained 4% (*w*/*v*) polyvinylpyrrolidone (PVP), 5 mM β-mercaptoethanol, and 2 mM ethylenediaminetetraacetic acid (EDTA); and then centrifuged at 12,000× *g* for 15 min at 4 °C. For the enzymatic reaction, 0.5 mL of the enzyme extract was mixed with 2.5 mL of 10 mM L-phenylalanine solution and incubated at 37 °C for 30 min. The addition of 0.5 mL of 6 M HCl resulted in the termination of the reaction. Subsequently, the absorbance was measured at 290 nm. The activity of PAL was quantified in terms of units per gram fresh weight (U·g^−1^), with one unit (U) defined as a change of 0.01 ΔA_290_ nm per minute.

Cinnamate-4-hydroxylase (C4H) was measured utilizing a slightly modified approach reported by Zhou et al. [22]. Fresh fruit tissue (1.0 g) was homogenized in 10 mL of ice-cold extraction buffer consisting of 50 mM Tris-HCl (adjusted to pH 8.9), 15 mM β-mercaptoethanol, 4 mM magnesium chloride (MgCl_2_), 5 mM ascorbic acid, 1 mM PMSF, 10% (*v*/*v*) glycerol, and 0.1 g PVP to stabilize phenolic interactions. The homogenate was then centrifuged at 12,000× *g* for 30 min at 4 °C, and the clarified supernatant was promptly used for enzymatic analysis. For the reaction, 0.8 mL of the enzyme extract was incubated with 2.5 mL of 50 mM Tris-HCl buffer and maintained at (pH 8.9 and a temperature of 25 °C for 30 min. The reaction was halted by the addition of 100 μL of 6 M HCl. C4H activity was quantified by measuring absorbance at 340 nm. Enzyme activity was expressed in terms of units per gram fresh weight (U·g^−1^), where one unit (U) corresponds to a 0.01 increase in absorbance at 340 nm per minute.

4-Coumarate-CoA ligase (4CL) was conducted grounded in the method described by Ji et al. [23] with slight modifications incorporated. Fresh fruit samples (1.0 g) were homogenized in 10 mL of 0.05 M Tris-HCl buffer (pH 8.0) containing 0.1 g of PVP. The homogenate was centrifuged at 12,000× *g* for 30 min at 4 °C, and the supernatant was obtained for subsequent enzyme activity assays. The reaction mixture was the combination of 0.5 mL of enzyme extract, 2 mL of 5 mM MgCl_2_, 0.5 mL of 5 mM adenosine triphosphate (ATP), 0.05 mL of 0.4 mM coenzyme A (CoA), and 0.05 mL of 0.6 mM *p*-coumaric acid. The mixture was incubated at 40 °C for 10 min, after which the reaction was terminated by cooling on ice. The determination of 4CL activity necessitated absorbance monitored at 333 nm. Enzyme activity, normalized to per gram fresh weight (U·g^−1^), was determined with one unit (U) defined as the rate of 0.01 ΔA_333_ nm per minute.

Chalcone synthase (CHS) activity was measured based on a protocol adapted from Ghasemzadeh et al. [24] with certain modifications. Fresh tissue (1.0 g) was homogenized in 10 mL of 100 mM borate buffer (pH 8.8) containing 1 mM 2-mercaptoethanol. The homogenate was centrifuged at 12,000× *g* for 30 min at 4 °C, and the supernatant was collected for subsequent enzyme activity assays. For the reaction, 0.1 mL of the enzyme extract was mixed with 1.89 mL of 100 mM Tris-HCl buffer (pH 7.8) that included 10 mM potassium cyanide. The mixture was further supplemented with 10 μL of ethylene glycol monomethyl ether containing 1 mg mL^−1^ chalcone and incubated at 30 °C for 5 min. CHS activity was determined in the absorbance at 371 nm. Enzyme activity was expressed as units per gram fresh weight (U·g^−1^), where one unit (U) was defined as 0.01 ΔA_371_ nm per minute.

Chalcone isomerase (CHI) activity was determined according to the previous study conducted by Wang et al. [25]. Briefly, frozen tissue samples (1.0 g) were homogenized in 10 mL of ice-cold 50 mM potassium phosphate buffer (pH 8.8) with 18 mM β-mercaptoethanol and 50 mM sodium ascorbate as antioxidants. The homogenate underwent centrifugation at 12,000× *g* for 30 min at 4 °C, and the resulting supernatant was collected for further enzymatic analysis. For the assay, 200 µL of the supernatant was supplemented into 2.2 mL of 50 mM Tris-HCl buffer (pH 7.4) containing 7.5 mg·mL^−1^ bovine serum albumin (BSA) and 250 mg L^−1^ sodium sulfite (Na_2_SO_3_). The reaction was initiated through the addition of 50 µL of 1.0 g L^−1^ 2’,4,4’,6’-tetrahydroxychalcone, followed by incubation at 34 °C for 30 min. The assessment of CHI activity involved monitoring the absorbance at 381 nm. The activity level of the enzyme, expressed as units per gram fresh weight (U gx^−1^), was standardized with one unit (U) equivalent to 0.01 ΔA_371_ nm per minute.

### 2.6. RNA Extraction, cDNA Synthesis, and qRT-PCR Analysis

Adhering to the manufacturer’s protocol, total RNA was extracted from pepper fruits using the Tiangen RNAprep Pure Plant Plus Kit (Tiangen Biochemical Technology Co., Ltd., Beijing, China). In addition, the synthesis of complementary DNA (cDNA) was achieved through reverse transcription under the manufacturer’s instructions. For real-time quantitative PCR (RT-qPCR) analysis, reactions were performed with SuperReal PreMix Plus SYBR Green Master Mix (Tiangen) on a QuantStudio5 Real-Time PCR System (Thermo Fisher Scientific, Wilmington, DE, USA). The reaction mixture (20 μL total volume) consisted of 2 μL cDNA template, 0.4 μL each of forward and reverse primers, 10 μL SYBR Green Master Mix, and 7.2 μL nuclease-free dd H_2_O. The primer sequences were designed using the National Center for Biotechnology Information (NCBI) database, as presented in Table 2. The CaActin gene was employed as an internal reference for normalization, and relative gene expression was calculated using the comparative 2^−ΔΔCt^ method.

### 2.7. Statistical Analysis

Data were analyzed using one-way analysis of variance (ANOVA) with SPSS software (version 22.0; SPSS Institute Inc., Chicago, IL, USA), and significant differences were compared using Duncan’s multiple range test (*p* < 0.05). Data were expressed as mean ± standard error (SE). Figures were made using Origin 2022 (Origin, Inc., San Francisco, CA, USA).

## 3. Results

### 3.1. Effect of Exogenous MT on the Contents of Total Phenolics and Total Flavonoids in Pepper Fruits

**Figure 2:**
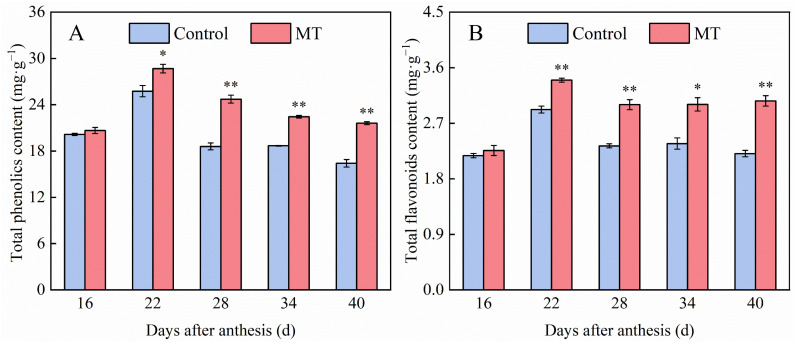
Effect of exogenous MT on the contents of total phenolics (**A**) and total flavonoids (**B**) in pepper fruits. The data are presented as the mean ± SE of three biological replicates. Asterisks * and **, respectively, denote apparent differences between the control and MT treatment fruits at *p* < 0.05 and *p* < 0.01.

In plants, total phenolics and flavonoids are central to phenylpropane metabolic pathways. As shown in Figure 2A, the total phenolics content in both control and MT treatment groups pepper fruits exhibited a consistent pattern of initial increase followed by gradual decrease during pepper ripening. Meanwhile, the total phenolics content of the MT treatment group was higher than that of the control group in all cases, especially at 22, 28, 34, and 40 days after anthesis, at which time point the contents increased by 11.35%, 32.95%, 20.15%, and 31.66%, respectively, when compared with those in the control group during the same period. As shown in Figure 2B, the total flavonoids content of the control and MT treatment groups showed similar trends, peaking at 22 days after anthesis and then remaining stable at the late stage of pepper ripening. At 22, 28, 34, and 40 days after anthesis, the total flavonoids contents in the MT treatment group were significantly higher than those in the control group at the same time point by 16.29%, 28.59%, 26.75%, and 38.55%, respectively.

### 3.2. Effect of Exogenous MT on the Contents of Phenolic Compounds in Pepper Fruits

**Figure 3:**
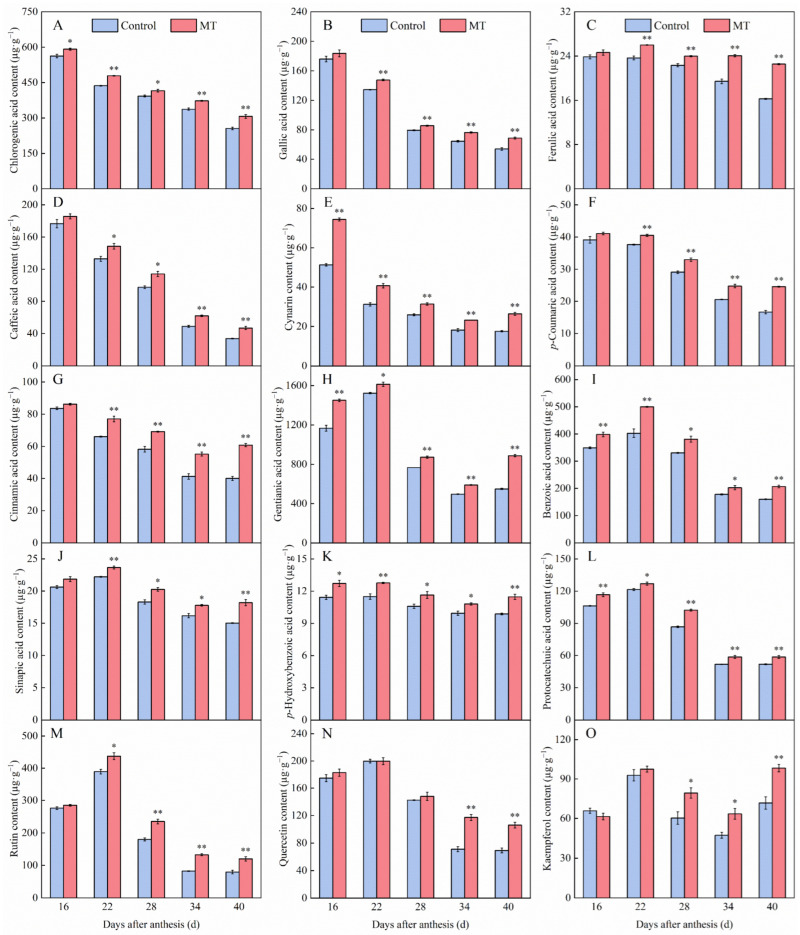
Effect of exogenous MT on the contents of chlorogenic acid (**A**), gallic acid (**B**), ferulic acid (**C**), caffeic acid (**D**), cynarin (**E**), *p*-coumaric acid (**F**), cinnamic acid (**G**), gentianic acid (**H**), benzoic acid (**I**), sinapic acid (**J**), *p*-hydroxybenzoic acid (**K**), protocatechuic acid (**L**), rutin (**M**), quercetin (**N**), and kaempferol (**O**) in pepper fruits. The data are presented as the mean ± SE of three biological replicates. Asterisks * and **, respectively, denote apparent differences between the control and MT treatment fruits at *p* < 0.05 and *p* < 0.01.

The contents of 12 phenolic acids (chlorogenic acid, gallic acid, ferulic acid, caffeic acid, cynarin, *p*-coumaric acid, cinnamic acid, gentianic acid, benzoic acid, sinapic acid, *p*-hydroxybenzoic acid, and protocatechuic acid), as well as three flavonoids (rutin, quercetin, and kaempferol), were determined to further investigate the effect of exogenous MT on phenolic compounds in pepper fruits. As shown in Figure 3, all 15 phenolic compounds were increased to different degrees after treatment with MT. Among the 12 phenolic acids, gentianic acid and chlorogenic acid were most abundant, reaching 1614.81 ± 21.33 and 591.71 ± 4.47 μg∙g^−1^, respectively, and the least abundant phenolic acid was *p*-hydroxybenzoic acid (10.81 ± 0.11 μg∙g^−1^). Among the three flavonoids, rutin and quercetin were most abundant, with values of 437.61 ± 10.60 and 199.78 ± 5.13 μg∙g^−1^, respectively, whereas kaempferol was least abundant (61.47 ± 2.49 μg∙g^−1^). Figure 3A–G demonstrate that the contents of chlorogenic acid, gallic acid, ferulic acid, caffeic acid, cynarin, *p*-coumaric acid, and cinnamic acid in both the control and MT treatment groups decreased progressively during pepper ripening. At 40 days after anthesis, the MT treatment group exhibited significantly higher levels of these phenolic compounds compared to the control group, with increases of 36.90% (chlorogenic acid), 27.29% (gallic acid), 38.62% (ferulic acid), 38.94% (caffeic acid), 50.34% (cynarin), 47.56% (*p*-coumaric acid), and 51.60% (cinnamic acid), respectively. In contrast, as shown in Figure 3H–L, in the control and MT treatment groups, gentianic acid, benzoic acid, sinapic acid, *p*-hydroxybenzoic acid, and protocatechuic acid peaked 22 days after anthesis and then decreased until pepper ripening. At 40 days after anthesis, MT treatment pepper fruits exhibited significantly higher levels of gentianic acid (61.70%), benzoic acid (29.34%), erucic acid (21.07%), *p*-hydroxybenzoic acid (23.23%), and protocatechuic acid (24.59%) compared to the control group. Figure 3M,N reveal similar trends in rutin and quercetin contents between the control and MT treatment pepper fruits. At 34 and 40 days after anthesis, the MT treatment group showed significant increases in rutin (60.84% and 51.16%, respectively) and quercetin (64.83% and 53.21%, respectively) compared to the control. In contrast, kaempferol content exhibited fluctuating trends in both groups (Figure 3O). By 40 days after anthesis, kaempferol levels in the MT treatment pepper fruits were 37.20% higher than those in the control.

**Figure 4:**
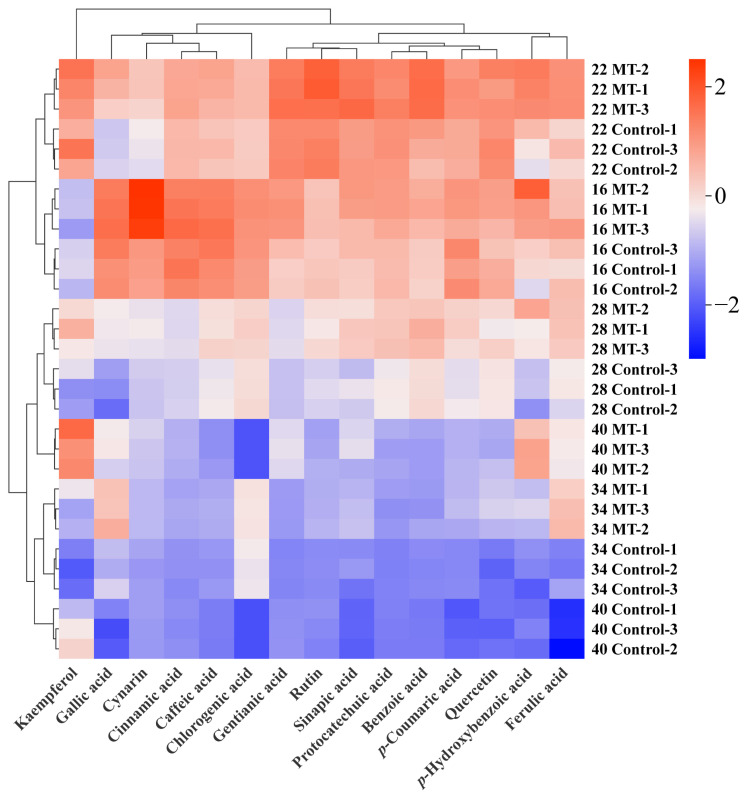
Cluster analysis of the phenolic compounds after exogenous MT treatment in pepper fruits. The colored block represents the relative values of the phenolic compounds in the corresponding position.

Furthermore, we performed a hierarchical clustering heatmap analysis of 15 phenolic compounds across different ripening stages. As shown in Figure 4, the contents of most phenolic compounds in the control and MT treatment groups reached their maximum at 22 days after anthesis and then began to decline until the pepper matured. MT treatment increased the contents of phenolic compounds in pepper fruits, especially during the late stages of fruit ripening. The 15 phenolic compounds were broadly classified into seven groups, as follows: (1) kaempferol was clustered into one class; (2) gallic acid and chlorogenic acid were clustered into one class; (3) cynarin, cinnamic acid and caffeic acid were clustered into one class. (4) gentianic acid, rutin, and sinapic acid were clustered into one class; (5) protocatechuic acid and benzoic acid were clustered into one class; (6) *p*-coumaric acid and quercetin were clustered into one class; and (7) *p*-hydroxybenzoic acid and ferulic acid were clustered into one class. This clustering pattern suggests potential synergies in the biosynthetic pathways or regulatory mechanisms among the grouped compounds during fruit maturation. The differential responses of these clusters to MT treatment provide valuable insights into the specific metabolic pathways influenced by this compound during pepper ripening.

### 3.3. Effect of Exogenous MT on Enzyme Activities Involved in the Phenylpropane Metabolism in Pepper Fruits

**Figure 5:**
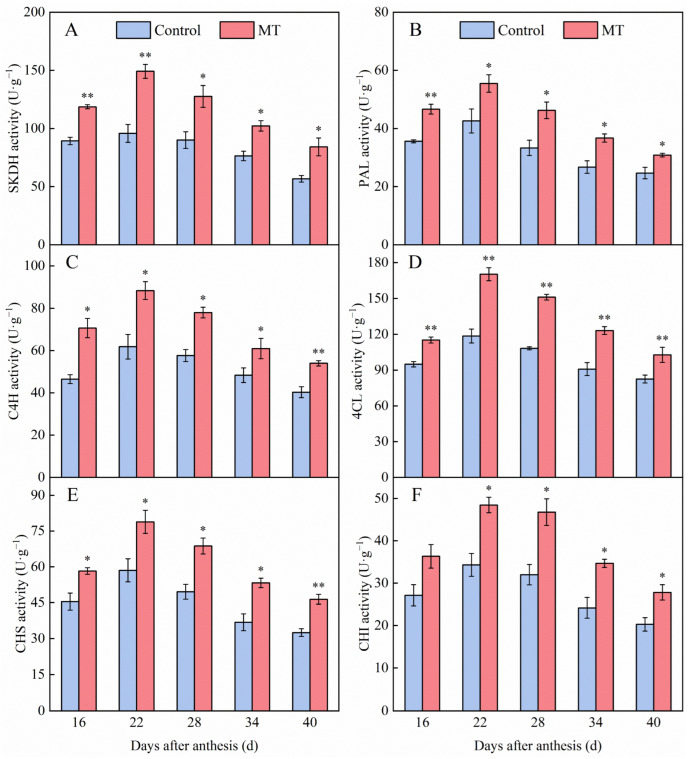
Effect of exogenous MT on the enzyme activities of SKDH (**A**), PAL (**B**), C4H (**C**), 4CL (**D**), CHS (**E**), CHI (**F**) involved in the phenylpropane metabolism in pepper fruits. The data are presented as the mean ± SE of three biological replicates. Asterisks * and **, respectively, denote apparent differences between the control and MT treatment fruits at *p* < 0.05 and *p* < 0.01.

To further investigate the effect of MT treatment on phenolic compounds metabolism in pepper fruits, the activities of six enzymes involved in phenolic compounds metabolic pathways were analyzed (Figure 5). Shikimate dehydrogenase (SKDH) regulates substrate availability for phenylpropane metabolism and indirectly modulates phenolic compound synthesis. As shown in Figure 5A, SKDH activity in the control and MT treatment groups first increased and then decreased during pepper ripening. Moreover, MT treatment significantly increased SKDH activity in pepper fruits 16, 22, 28, 34, and 40 days after anthesis, with SKDH activities in MT treatment fruits being 1.33-, 1.56-, 1.42-, 1.34-, and 1.48-fold higher than those in control pepper fruits, respectively.

Phenylalanine ammonia-lyase (PAL), cinnamate-4-hydroxylase (C4H), and 4-coumarate-CoA ligase (4CL) are three key enzymes involved in the phenylpropane metabolic pathway. As illustrated in Figure 5B, PAL activity in both control and MT treatment groups displayed comparable developmental patterns, peaking at 22 days after anthesis before declining until fruit maturity. Notably, MT application significantly enhanced PAL activity, particularly at 28 days after anthesis when the treated fruits exhibited 1.39-fold higher PAL activity compared to control. This elevation persisted throughout the ripening phase, with MT treatment fruits consistently maintaining superior PAL levels. Figure 5C,D demonstrate that C4H and 4CL activities followed analogous trajectories in both experimental groups. MT treatment markedly boosted both enzymatic activities, reaching peak values at 22 days after anthesis with 1.43- and 1.44-fold increases over control levels for C4H and 4CL, respectively. 

Flavonoids are a class of secondary metabolites with antioxidant and antibacterial properties that can bind to and neutralize harmful free radicals. Chalcone synthase (CHS) and chalcone isomerase (CHI) are two key rate-limiting enzymes in the flavonoid synthesis pathway. As depicted in Figure 5E, MT treatment pepper fruits exhibited consistently higher CHS activity compared to controls throughout ripening. The most pronounced difference occurred at 34 days after anthesis days after anthesis, when CHS activity in MT treatment fruits reached 1.45-fold of the control level (1.45 times the control value). Similarly, Figure 5F demonstrates that CHI activity in both groups peaked at 22 days after anthesis before gradually declining until full fruit maturation. Notably, MT treatment peppers maintained significantly elevated CHI activity relative to controls across all timepoints, with the maximum differential (1.46-fold higher than control) observed at 28 days after anthesis.

### 3.4. Effect of Exogenous MT on Genes Expression Involved in the Phenylpropane Metabolism in Pepper Fruits

**Figure 6:**
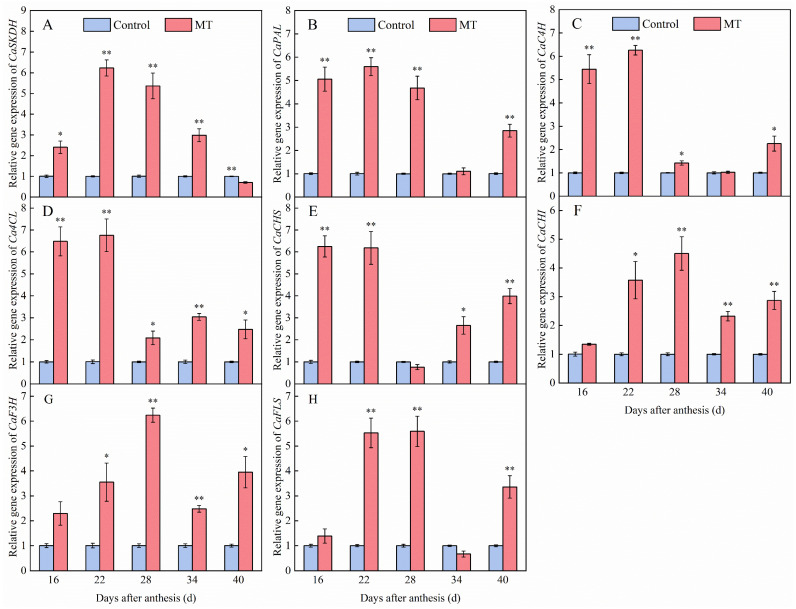
Effect of exogenous MT on the expression of *CaSKDH* (**A**), *CaPAL* (**B**), *CaC4H* (**C**), *Ca4CL* (**D**), *CaCHS* (**E**), *CaCHI* (**F**), *CaF3H* (**G**), and *CaFLS* (**H**) genes involved in the phenylpropane metabolism in pepper fruits. The data are presented as the mean ± SE of three biological replicates. Asterisks * and **, respectively, denote apparent differences between the control and MT treatment fruits at *p* < 0.05 and *p* < 0.01.

To further investigate the molecular mechanisms underlying the effects of MT treatment on phenolic compounds synthesis in pepper fruits, the expression of genes related to this pathway, including *CaSKDH*, *CaPAL*, *CaC4H*, *CaC4CL*, *CaCHS*, *CaCHI*, *CaF3H*, and *CaFLS*, was determined. As shown in Figure 6, compared with that in the control, MT treatment upregulated *CaSKDH* expression in pepper fruits at 16, 22, 28, and 34 days after anthesis, and this was 2.40-, 6.23-, 2.35-, and 2.98-fold higher than that in the control pepper fruits, respectively, whereas expression was significantly downregulated at 40 days after anthesis (Figure 6A). Further, MT treatment upregulated the expression of *CaPAL*, *CaC4H*, and *Ca4CL* during pepper ripening, and the levels of all of these genes reached a maximum at 22 days after anthesis, showing increases of 5.57-, 6.25-, and 6.71-fold, respectively, when compared with those in control pepper fruits at the same time (Figure 6B–D). Compared to that in the control group, MT treatment significantly upregulated *CaCHS* expression at 16, 22, 34, and 40 days after anthesis, and this was 6.22-, 6.18-, 2.64-, and 3.98-fold higher, respectively, compared to that in control pepper fruits during the same period. In contrast, it downregulated the expression of this gene 28 days after anthesis (Figure 6E). Meanwhile, 100 μmol∙L^−1^ MT treatment upregulated *CaCHI* expression at each flowering time point, and this reached its maximum 28 days after anthesis, at which time it was 4.49 times higher than that in the control group (Figure 6F). Exogenous MT treatment also upregulated *CaF3H* expression levels during pepper ripening, especially at 22, 28, 34, and 40 days after anthesis, at which time it was increased by 3.52-, 6.21-, 2.49-, and 3.92-fold, respectively, compared to that in the control group (Figure 6G). Further, MT treatment significantly upregulated *CaFLS* expression at 22, 28, and 40 days after anthesis, and this was 5.52-, 5.57-, and 3.35-fold higher than that in the control pepper fruits at the same times, but it downregulated *CaFLS* expression at 34 days after anthesis (Figure 6H).

### 3.5. Effect of Exogenous MT on the Content of Exogenous MT in Pepper Fruits

**Figure 7:**
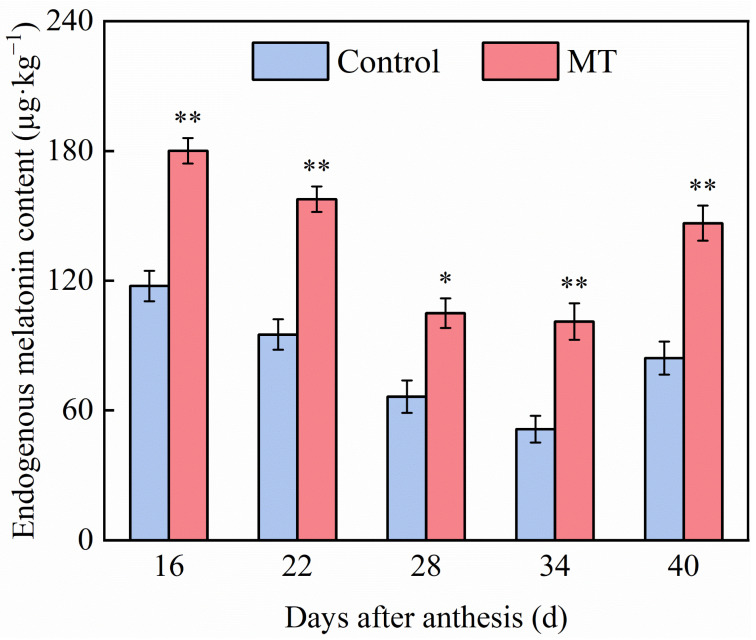
Effect of exogenous MT on the content of endogenous MT in pepper fruits. The data are presented as the mean ± SE of three biological replicates. Asterisks * and **, respectively, denote apparent differences between the control and MT treatment fruits at *p* < 0.05 and *p* < 0.01.

The effect of exogenous MT on the endogenous MT content during pepper ripening is shown in Figure 7. The endogenous MT content of both control and MT treatment groups showed a decreasing and then increasing trend. Compared with that in control at the same time points, 100 μmol∙L^−1^ MT treatment significantly increased the endogenous MT content in pepper fruits at 16, 22, 28, 34, and 40 days after anthesis by 53.27%, 65.85%, 58.17%, 97.00%, and 74.23%, respectively.

### 3.6. Effect of Exogenous MT on Genes Expression Involved in the Endogenous MT Synthesis Pathway in Pepper Fruits

**Figure 8:**
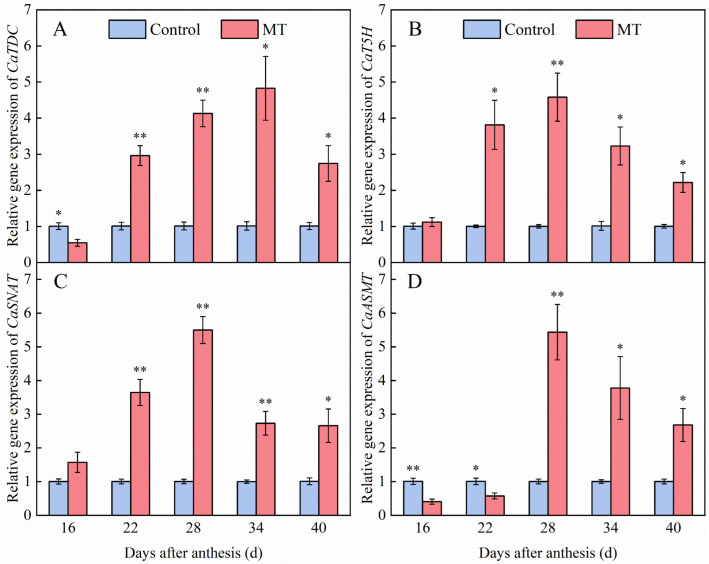
Effect of exogenous MT on the expression of *CaTDC* (**A**), *CaT5H* (**B**), *CaSNAT* (**C**), and *CaASMT* (**D**) genes involved in the endogenous MT synthesis pathway in pepper fruits. The data are presented as the mean ± SE of three biological replicates. Asterisks * and **, respectively, denote apparent differences between the control and MT treatment fruits at *p* < 0.05 and *p* < 0.01.

The expression levels of four genes involved in endogenous MT biosynthesis, namely *CaTDC*, *CaT5H*, *CaSNAT*, and *CaASMT*, were measured to further investigate the effect of exogenous MT on the endogenous MT-synthesis mechanism during pepper ripening. The expression of *CaTDC* after treatment with 100 μmol·L^−1^ gradually increased, peaking at 34 days after anthesis, exhibiting 4.76-fold higher relative expression compared to that in the control group (Figure 8A). In the MT treatment fruits, *CaT5H* expression initially increased and then decreased during ripening. However, its expression remained consistently higher than that in the control group at all stages. Further, significant upregulation of expression was observed at 22, 28, 34, and 40 days after anthesis, with fold-changes of 3.81, 4.57, 3.18, and 2.21, respectively (Figure 8B). *CaSNAT* expression after MT treatment was consistently elevated compared with that in the control group throughout fruit ripening. Specifically, significant increases (3.81-, 4.57-, 3.18-, and 2.21-fold, respectively) were observed at 22, 28, 34, and 40 days after anthesis (Figure 8C). As shown in Figure 8D, MT treatment significantly upregulated *CaASMT* expression at 28, 34, and 40 days after anthesis, with expression levels being 5.41-, 3.77-, and 2.67-fold higher than those in the control group, respectively. However, *CaASMT* expression was significantly downregulated 16 and 22 days after anthesis. This transcriptional pattern explains the observed 97% endogenous MT increase at 34 days after anthesis, demonstrating the dual role of MT as both a precursor and pathway regulator in pepper fruits metabolism.

### 3.7. Correlation Analysis

**Figure 9:**
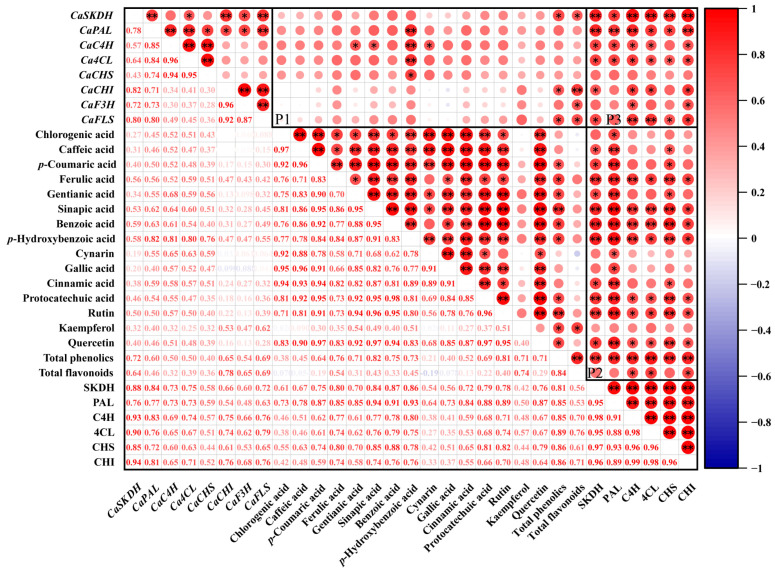
Correlation analysis between genes expression, phenolic compounds contents, and enzyme activities in pepper fruits. Pearson correlation coefficients. Red (+1) and blue (−1) colors indicate positive and negative correlations between the different indices, respectively. The * and ** represent significant correlations at the *p* < 0.05 and *p* < 0.01 levels, respectively.

To get a better insight into the role of exogenous MT in phenolic compounds contents of pepper during ripening, correlation analysis between genes expression, phenolic compounds contents and enzymatic activities in MT treatment pepper fruits were performed and the graph was conducted according to Pearson’s correlation coefficients (r). The P1, P2, and P3 regions, shown in the Figure 9, indicate the correlations between genes expression and phenolic compounds contents, phenolic compounds and enzymatic activities, as well as genes expression and enzymatic activities, respectively.

In the P1 region, *CaSKDH* expression was positively correlated with benzoic acid, *p*-hydroxybenzoic acid, ferulic acid, sinapic acid, and rutin (0.50 ≤ r^2^ ≤ 0.59), whereas the correlation coefficients with other phenolic compounds were relatively low. *CaPAL* expression was positively correlated with benzoic acid, *p*-hydroxybenzoic acid, and sinapic acid (0.62 ≤ r^2^ ≤ 0.82), whereas the correlation coefficients with other phenolic compounds were relatively low. *CaC4H* expression was positively correlated with *p*-hydroxybenzoic acid, gentisic acid, cynarin, sinapic acid, and benzoic acid (0.61 ≤ r^2^ ≤ 0.81), whereas the correlation coefficients with other phenolic compounds were relatively low. *Ca4CL* expression was positively correlated with *p*-hydroxybenzoic, cynarin, and sinapic acid (0.60 ≤ r^2^ ≤ 0.80), but the correlation coefficients with other phenolic compounds were relatively low. *CaCHS* expression was positively correlated with *p*-hydroxybenzoic acid (r^2^ = 0.76) with relatively low correlation coefficients with other phenolic compounds. *CaCHI* expression showed a negative correlation with cynarin and gallic acid (−0.1 ≤ r^2^ ≤ −0.03) and a positive correlation with other phenolic compounds, though the correlation coefficients were relatively low (0 ≤ r^2^ ≤ 0.53). Similarly, *CaF3H* expression showed a negative correlation with gallic acid (r^2^ = −0.09) and a positive correlation with other phenolic compounds, but the correlation coefficients were also relatively low (0.04 ≤ r^2^ ≤ 0.47). *CaFLS* expression was positively correlated with phenolic compounds, but the correlation coefficient was relatively low (0.04 ≤ r^2^ ≤ 0.69).

In the P2 region, the activities of SKDH, PAL, C4H, 4CL, CHS, and CHI enzymes showed positive correlations with most phenolic compounds, exhibiting relatively high correlation coefficients with, sinapic acid, benzoic acid, *p*-hydroxybenzoic acid, ferulic acid, quercetin, protocatechuic acid, and rutin (0.61 ≤ r^2^ ≤ 0.94). In contrast, the correlation coefficients between the enzyme activity and other phenolic compounds were relatively low. In the P3 region, the relative expression of *CaSKDH*, *CaPAL*, *CaC4H*, *Ca4CL*, *CaCHS*, *CaCHI*, *CaF3H*, and *CaFLS* was positively correlated with all the activities of all enzymes (0.44 ≤ r^2^ ≤ 0.94). Notably, higher correlation coefficients (0.60 ≤ r^2^ ≤ 0.94) were observed between the relative expression of *CaSKDH*, *CaPAL*, *CaC4H*, *Ca4CL*, and *CaFLS* and enzyme activities.

## 4. Discussion

Phenylpropane metabolism is central to plant secondary metabolism, and its products, including phenolic compounds, flavonoids, and lignans, serve as the structural components of plants and have significant biological activities [26]. These compounds play vital roles in enhancing plant resistance to diseases and stress [27]. In addition, these compounds are intricately linked to the coloration, quality, and flavor of fruits and vegetables, which have an important effect on their nutritional value [28]. In the present study, MT increased the contents of total phenolics and flavonoids, which was in accordance with previous studies on strawberries [29], blueberries [30], plums [31], and grapes [32], in which the total phenolics and flavonoids contents increased after MT application. It is well known that the phenolic compounds in pepper consist mainly of caffeic acid, ferulic acid, sinapic acid, vanillic acid, *p*-coumaric acid, and *p*-hydroxybenzoic acid [33]. Ferulic acid has strong anti-radical properties [34], and vanillic acid is primarily used as a flavoring enhancer [35]. It has also been reported that flavonoids are the main phenolic compounds in peppers [33]. Previous studies have shown that most flavonoids found in peppers are glycosides and aglycones of myricetin, quercetin, luteolin, apigenin, and kaempferol [36]. As previously reported, the synergistic effects of the various phenolic compounds in pepper fruits confer antioxidant properties to the pepper [37,38,39]. In addition, these compounds not only give peppers their distinctive flavor and pungency but also have medicinal properties, such as cancer and atherosclerosis prevention and anti-inflammatory activity [40]. In addition, MT promoted the phenolic compounds contents in pepper during the ripening period, consistent with previous findings in bananas [41], pears [42], jujubes [43], and tomatoes [44], indicating that this compound could have an important role in regulating the accumulation of phenolic compounds. Low temperatures detract from the flavor and nutritional quality of fruits, making the application of antioxidants to increase the total phenolics content crucial for improving fruits quality and resistance [45]. Notably, this study also found that the contents of most phenolic compounds in pepper fruits increased at 22 days post-flowering, indicating that low-temperature stress induced the synthesis of phenols, potentially contributing to the cold-resistance response of fruits as antioxidants. MT treatment increased the contents of phenolic compounds in pepper fruits and improved or maintained the quality of the pepper fruits.

The shikimate pathway comprises a central stage in the biosynthesis of aromatic amino acids (i.e., tyrosine, phenylalanine, and tryptophan) and specialized metabolites in plants and is a vital conduit between glucose metabolism and secondary metabolism [46]. In the plant shikimate pathway, shikimate dehydrogenase (SKDH) catalyzes the synthesis of shikimate-derived phenylalanine, leading to the synthesis of salicylic acid, furan coumarin, chlorogenic acid, lignin, stilbene, proanthocyanidins, flavonoids (including flavanones, flavanols, and anthocyanins), and isoflavones through multiple enzymatic reactions [47]. In this study, MT treatment markedly increased SKDH activity in pepper fruits and accelerated the production of phenylalanine, providing the foundation for phenylpropane metabolism. As a key rate-limiting enzyme in the first step of the propane metabolic pathway, phenylalanine ammonia-lyase (PAL) catalyzes the conversion of phenylalanine to trans-cinnamic acid, thereby initiating the entire phenylpropanoid metabolic pathway [48]. In the next stage, cinnamate-4-hydroxylase (C4H), a significant node enzyme in the phenylpropanoid metabolic pathway, facilitates the transformation of cinnamic acid into *p*-coumaric acid, which is then introduced into the branched pathway [49]. 4-Coumarate-CoA ligase (4CL) represents a branch point in the phenylpropanoid metabolic pathway that directs downstream metabolism to produce lignans or flavonoids [50]. Chalcone synthase (CHS) is widely distributed in plants and is the first key enzyme in the flavonoid biosynthetic pathway; in addition, it can catalyze reactions involving *p*-coumaroyl-CoA and malonyl-CoA for the generation of naringenin chalcone, which is derived from the isomerization of chalcone isomerase (CHI) to other flavonoids [51]. The regulation of these enzyme activities is beneficial for augmenting disease resistance and fruits quality [52,53,54]. One study showed that exogenous MT application improved the activities of PAL, C4H, and 4CL enzymes in phenylpropane metabolism in litchi fruits, which not only promoted the accumulation of total phenolics and flavonoids but also resulted in the inhibition of downy blight and enhancement of resistance [55]. MT treatment also enhances phenolics, flavonoids, and anthocyanin contents, delays cherry senescence, and increases the antioxidant potential during cold storage by enhancing PAL and CHS activities and inhibiting polyphenol oxidase activity [56]. Similarly, MT increases PAL, 4CL, and 4CH enzyme activities by activating the phenylpropanoid pathway, maintaining plum quality, and extending its shelf life [31]. Consistent with these findings, our experiment showed that, compared with those in the control group, exogenous MT promoted the activities of key enzymes involved in phenylpropane metabolism (SKDH, PAL, 4CL, C4H, CHS, and CHI) during pepper ripening, which was accompanied by elevated levels of total phenolics, total flavonoids, and the main individual phenolic compounds. These results are consistent with the positive correlations among total phenolics, total flavonoids, and most of the individual phenolic compounds and enzyme activities, indicating that MT may promote the biosynthesis of phenolic compounds via the induction of enzyme activities related to the phenylpropanoid pathway. Furthermore, we explored the mRNA levels of genes involved in phenolic compounds biosynthesis. MT treatment was found to accelerate strawberry ripening by increasing *FaPAL* and *FaCHS* expression and enzyme activity, thereby promoting the accumulation of phenols and anthocyanins [57]. In addition, the application of MT increased the levels of phenolic compounds in date fruits while upregulating the expression of *ZjPAL*, *ZjC4H*, and *ZjCHS*, the main genes involved in phenolic compounds biosynthesis, delaying post-harvest senescence and maintaining quality attributes [43]. Similarly, MT treatment has been shown to upregulate the expression of the antioxidant-encoding genes *VcPAL*, *VcAPX*, and *VcGST*, which improves antioxidant activity to a certain extent anddelays the senescence of blueberry fruits [30]. Our study demonstrated that the expression of *CaSKDH*, *CaPAL*, *CaC4H*, *Ca4CL*, *CaCHS*, *CaCHI*, *CaF3H*, and *CaFLS* were upregulated after exogenous MT treatment, corresponding to the increased content of the aforementioned compounds (Figure 3). Collectively, these results suggest that MT plays a vital role in promoting the regulation of genes involved in the phenylpropanoid pathway.

As a key signaling molecule, phytomelatonin exerts a significant effect on the regulation of the synthesis and metabolism of a variety of endogenous hormones, as well as their associated signaling pathways, ultimately influencing plant growth, development, and responses to adversity [58]. As expected, exogenous MT treatment significantly enhanced endogenous MT content during pepper ripening, which was consistent with the results of previous studies on peaches [19], tomatoes [59], and cherries [60], showing that the endogenous MT content increased after MT application. Previous studies have shown that exogenous MT stimulates endogenous MT biosynthesis by upregulating the expression of related genes. It was found that 100 μmol∙L^−1^ MT treatment significantly upregulated the expression of key genes involved in MT synthesis, such as *FaTDC*, *FaT5H*, *FaSNAT*, and *FaASMT*, in strawberries, leading to increased endogenous MT accumulation [61]. In addition, exogenous MT treatment markedly upregulates the transcription levels of MT biosynthesis genes, such as *PaTDC*, *PaT5H*, *PaASMT*, and *PaSNAT*, which correlates with changes in MT contents in cherry [62]. Here, MT application upregulated the expression of key genes involved in the endogenous MT synthesis pathway, namely *CaTDC*, *CaT5H*, *CaASMT*, and *CaSNAT*, thereby promoting endogenous MT synthesis. Consequently, in this study, it was hypothesized that one of the mechanisms by which MT treatment improves fruits quality may involve upregulation of the expression of key genes involved in the endogenous MT anabolic pathway. This, in turn, may promote the accumulation of endogenous MT and trigger a series of reactions, including antioxidant reactions, enzyme catalysis, and nutrient biosynthesis.

**Figure 10:**
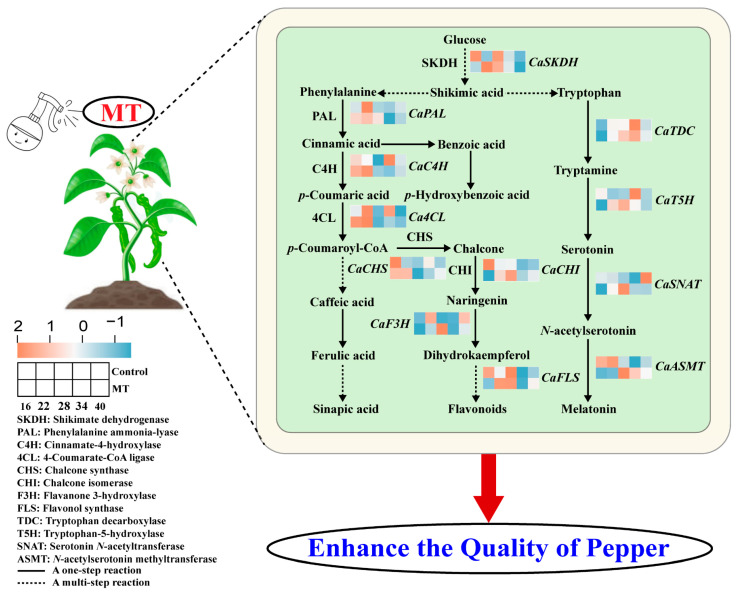
MT-mediated regulation of phenolic compounds synthesis pathways during pepper ripening.

Our experimental findings revealed that exogenous MT treatment significantly augmented phenolic compounds biosynthesis in pepper fruits through the regulation of gene expression and enzymatic activity. Indeed, correlation analyses revealed positive correlations among total phenolics, total flavonoids, and most phenolic compounds and overall enzyme activities and the expression of most genes, indicating that transcriptional and translational regulation are pivotal mechanisms through which MT regulates phenolic compounds biosynthesis (Figure 9). Furthermore, the data showed a self-sustaining metabolic loop mediated by exogenous MT in pepper fruits, as schematically illustrated in Figure 10. This regulatory cascade functions in three coordinated phases, as follows: (1) priming the shikimate–phenylpropanoid flux, (2) stimulating endogenous MT biosynthesis, and (3) creating an antioxidant synergy for sustained phytochemical production. This hormone–metabolic coupling positions MT as both a regulator and beneficiary of enhanced secondary metabolism in pepper fruits. This refined model maintains scientific rigor while enhancing clarity, effectively elucidating the role of MT as both a metabolic initiator and systemic stabilizer in phenolic compounds biosynthesis. Phenolic compounds have multiple functions, such as defense and stress resistance, promoting reproduction and dissemination, regulating band metabolism and energy allocation, environmental adaptation and signaling, and enhancing the nutritional and flavor qualities of fruits and vegetables, and these are closely related to environmental and human needs. The exogenous MT-induced increase in phenolic compounds is essentially a ‘fine-tuning’ strategy that can enhance resistance or quality by moderately activating the intrinsic metabolic network of the plant while avoiding excessive resource consumption or negative phenotypes.

## 5. Conclusions

Our results showed that exogenous MT enhances the enzymatic activities of SKDH, PAL, C4H, 4CL, CHS, and CHI, thereby enhancing the total phenolics, flavonoids, and phenolic compounds contents during pepper ripening. Moreover, the upregulation of the expression of key genes involved in phenolic compounds and endogenous MT metabolic pathways demonstrated the function of exogenous MT from a molecular perspective. This dual regulatory mechanism not only strengthens disease resistance and stress tolerance in pepper plants but also potentially improves fruits quality by enhancing the accumulation of secondary metabolites. This type of precise regulation provides a flexible and sustainable technological pathway for improving agricultural quality and efficiency, developing functional components, and studying plant resistance mechanisms. In the future, we need to further understand the molecular mechanism of melatonin in pepper fruits to lay a solid foundation for quality improvement breeding. 

## Figures and Tables

**Table 1:** Detection of different phenolic compounds by HPLC at different wavelengths.

Wavelength	240 nm	280 nm	322 nm
Phenolic compounds	Rutin	Benzoic acid	Gentianic acid
	Protocatechuic acid	Ferulic acid	Sinapic acid
	Quercetin	Cinnamic acid	Cynarin
	*p*-Hydroxybenzoic acid	*p*-Coumaric acid	Kaempferol
	Chlorogenic acid	Gallic acid	Caffeic acid

**Table 2:** Gene primer sequences in fluorescence quantitative PCR.

Gene Name	Gene ID	5′-3′ of the Forward Primer	5′-3′ of the Reverse Primer
*CaSKDH*	XM_016721816	GCAGAGGTTGGAGCTACAGTTGTG	CAGGTGCTAATCCGTCGGTGAAC
*CaPAL*	XM_016699298	GGCGTCGATGGTCCTGTTTGAG	GTGTGTCAGATGGTCCGTGAACTC
*CaC4H*	NM_001325053	CGGTGAGCATTGGAGGAAGATGAG	CCTCAACAACACTAGCCACCTCAG
*Ca4CL*	NM_001324830	AGGGACTACAGGGCTACCAAAAGG	AAAGGCAAGCAACACATCAACACG
*CaCHS*	NM_001325005	ATGGTGACCGTGGAGGAGGTTC	GAAGGAGTCGCCGTGCCAATG
*CaCHI*	XM_047399431	CCGGCTGGGTCATTGACGATTAG	TCTTTGCGGCAGGTGAAACTCC
*CaF3H*	XM_016722868	TCATGCTTCCACCTGGCTCTG	GGAGAATAACTCTTCCATCACCTGTC
*CaFLS*	XM_016716195	GCCTAAGAACCCTCCTTCCTACAG	CCAATCCAAGCCCAAGTGACAAG
*CaTDC*	XM_016725244	GTGGATTACAAAGACTGGCAAATAGG	TAATGTGACTCTGAAGATTGGCTACG
*CaT5H*	XM_016717872	CTCAACTCGCCGAACTCATACTC	GTAATATCAGAGCAACCGAAGGAGAG
*CaSNAT*	XM_016697799	CCTCCATCACCTCAATTCCATCTTC	GCAAACGCTACAGGGCTTCC
*CaASMT*	XM_047403946	TGGTGGTGATGGAACAACATTAAGG	TGCAACAGAAGCAACATGAGGTAG
*CaACTIN*	XM_016720449	CTGCTGTCCATCTGCTCTCTGTTG	GTGGTCTCGTATTTGGCCCTGTC

## Data Availability

The data presented in this study are available on request from the corresponding author. The data are not publicly available due to privacy restrictions.
